# RETRACTION: Examining the Relationship Between Nutritional Status and Wound Healing in Head and Neck Cancer Treatment: A Focus on Malnutrition and Nutrient Deficiencies

**DOI:** 10.1111/iwj.70542

**Published:** 2025-04-18

## Abstract

**RETRACTION:** ChenY. , LiY. , ZengY. , LiC. , LiY. , WangY. , and WangK. , “Examining the Relationship Between Nutritional Status and Wound Healing in Head and Neck Cancer Treatment: A Focus on Malnutrition and Nutrient Deficiencies,” International Wound Journal 21, no. 3 (2024): e14810, 10.1111/iwj.14810.38414357 PMC10899863

The above article, published online on 27 February 2024, in Wiley Online Library (http://onlinelibrary.wiley.com/) and its correction (https://doi.org/10.1111/iwj.14876), has been retracted by agreement between the journal Editor in Chief, Professor Keith Harding; and John Wiley & Sons Ltd. Following an investigation by the publisher, all parties have concluded that this article was accepted solely on the basis of a compromised peer review process. The editors have therefore decided to retract the article. The authors disagree with the retraction.

**RETRACTION:** Y. Chen , Y. Li , Y. Zeng , C. Li , Y. Li , Y. Wang , and K. Wang , “Examining the Relationship Between Nutritional Status and Wound Healing in Head and Neck Cancer Treatment: A Focus on Malnutrition and Nutrient Deficiencies,” International Wound Journal 21, no. 3 (2024): e14810, 10.1111/iwj.14810.38414357 PMC10899863

The above article, published online on 27 February 2024, in Wiley Online Library (http://onlinelibrary.wiley.com/) and its correction (https://doi.org/10.1111/iwj.14876), has been retracted by agreement between the journal Editor in Chief, Professor Keith Harding; and John Wiley & Sons Ltd. Following an investigation by the publisher, all parties have concluded that this article was accepted solely on the basis of a compromised peer review process. The editors have therefore decided to retract the article. The authors disagree with the retraction.

